# Meeting Summary: Global Vaccine and Immunization Research Forum, 2021

**DOI:** 10.1016/j.vaccine.2023.02.028

**Published:** 2023-03-10

**Authors:** Andrew Ford, Angela Hwang, Annie X. Mo, Shahida Baqar, Nancy Touchette, Carolyn Deal, Deborah King, Kristen Earle, Birgitte Giersing, Peter Dull, B. Fenton Hall

**Affiliations:** a Division of Microbiology and Infectious Diseases, National Institute of Allergy and Infectious Diseases, National Institutes of Health, MSC 9825, Bethesda, MD 20892-9825, USA; b Angela Hwang Consulting, PO Box 6601, Albany, CA 94706, USA; c Office of Global Research, National Institute of Allergy and Infectious Diseases, National Institutes of Health, Bethesda, MD, USA; d Infectious Disease Health Challenge - Prevention, Wellcome Trust, London NW1 2BE, United Kingdom; e Vaccine Development & Surveillance, Bill & Melinda Gates Foundation, PO Box 23350, Seattle, Washington 98102, USA; f Department of Immunization, Vaccines, and Biologicals, World Health Organization, Geneva, Switzerland

**Keywords:** BCG, bacille Calmette-Guérin, bnAb, broadly neutralizing antibody, CEPI, Coalition for Epidemic Preparedness Innovations, CHIM, controlled human infection model, ECVP, Evidence Considerations for Vaccine Policy, EUA, Emergency Use Authorization, EUL, Emergency Use Listing, GVAP, Global Vaccine Action Plan, GVIRF, Global Vaccine and Immunization Research Forum, HIV, human immunodeficiency virus, HPV, human papillomavirus, IA2030, Immunization Agenda 2030, MERS, Middle East respiratory syndrome, nOPV-2, novel oral poliomyelitis type 2 vaccine, PPP, public–private partnership, R&D, research and development, SARS, severe acute respiratory syndrome, VIPS, Vaccine Innovation Prioritisation Strategy

## Abstract

The 2021 Global Vaccine and Immunization Research Forum highlighted the considerable advances and recent progress in research and development for vaccines and immunization, critically reviewed lessons learned from COVID-19 vaccine programs, and looked ahead to opportunities for this decade.

For COVID-19, decades of investments in basic and translational research, new technology platforms, and vaccines targeting prototype pathogens enabled a rapid, global response. Unprecedented global coordination and partnership have played an essential role in creating and delivering COVID-19 vaccines. More improvement is needed in product attributes such as deliverability, and in equitable access to vaccines.

Developments in other priority areas included: the halting of two human immunodeficiency virus vaccine trials due to lack of efficacy in preventing infection; promising efficacy results in Phase 2 trials of two tuberculosis vaccines; pilot implementation of the most advanced malaria vaccine candidate in three countries; trials of human papillomavirus vaccines given in single-dose regimens; and emergency use listing of a novel, oral poliomyelitis type 2 vaccine. More systematic, proactive approaches are being developed for fostering vaccine uptake and demand, aligning on priorities for investment by the public and private sectors, and accelerating policy making.

Participants emphasized that addressing endemic disease is intertwined with emergency preparedness and pandemic response, so that advances in one area create opportunities in the other. In this decade, advances made in response to the COVID-19 pandemic should accelerate availability of vaccines for other diseases, contribute to preparedness for future pandemics, and help to achieve impact and equity under Immunization Agenda 2030.

## Highlights

• Global collaboration and decades of vaccine research continue to yield benefits.
• Such investments and experience enabled a rapid response to COVID-19.
• Advances made due to COVID-19 should be applied to other vaccines and to emergency preparedness.

## 1 Introduction

Vaccines continue to be among the most cost-effective public health interventions, contributing to longer, healthier, and more productive lives [1]. They are particularly important in low-income countries, contributing to progress on broader issues such as poverty and inequity [2].

Sponsored every two years by the World Health Organization (WHO), the US National Institute of Allergy and Infectious Diseases (NIAID), and the Bill & Melinda Gates Foundation, the Global Vaccine and Immunization Research Forum (GVIRF) aims to accelerate progress in vaccine and immunization research by promoting end-to-end dialog among diverse stakeholders.

Past GVIRF meetings have reviewed progress in the research and development (R&D) components of the Global Vaccine Action Plan (GVAP). Held in February 2021, this GVIRF marked the transition from GVAP to its successor, the Immunization Agenda 2030 (IA2030) [3]. Delayed one year by the COVID-19 pandemic, GVIRF 2021 took stock of the recent progress in vaccine and immunization research, including progress against the pandemic. GVIRF participants also identified ways that advances made to combat COVID-19 can accelerate progress against other diseases and highlighted synergies between addressing endemic diseases and preparing for emergencies.

GVIRF 2021 was held as a virtual meeting, enabling participation by a wider audience than possible in prior, in-person GVIRF meetings. This report shares key messages conveyed during the meeting. In light of rapid change in vaccines and immunization, the content has been updated with key developments since the meeting. Full proceedings of GVIRF 2021 are available online at https://www.technet-21.org/en/topics/gvirf.

## 2 Essential success factors for COVID-19 vaccines

**Fig. 1:**
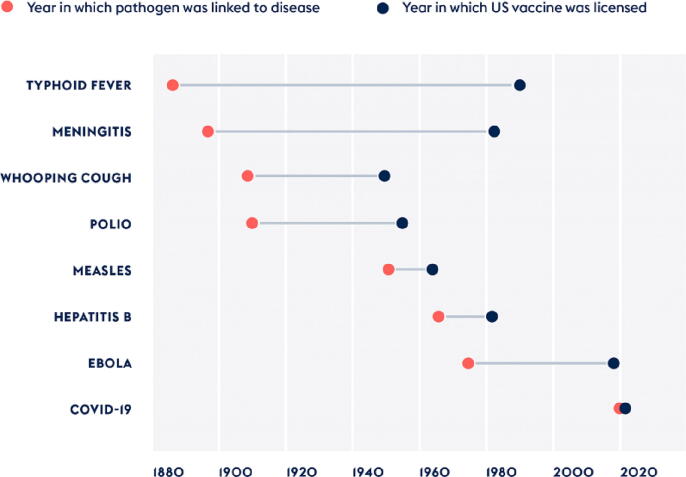
Rapid progress in vaccine innovation [4].

Past vaccines have taken decades from the identification and characterization of the responsible pathogens to the development and deployment of a new vaccine. Safe and effective COVID-19 vaccines were developed and made available with unprecedented speed (Fig. 1). During the COVID-19 pandemic, multiple vaccines were authorized for emergency use within a year after the initial identification of SARS-CoV-2, the virus responsible for COVID-19.

Vaccine development for COVID-19 is continuing in order to improve compatibility with existing immunization programs, especially for lower-resource settings. Priorities include improving thermostability, duration of protection, breadth of protection against SARS-CoV-2 variants, suitability for vulnerable populations (such as the immunocompromised), affordability, and other product attributes. Efforts are also underway to advance the development of broadly protective coronavirus vaccines [5], [6]. Participants at GVIRF discussed factors that have enabled progress to date.

**Basic research and early discovery** are essential foundations for success during and between emergencies. The rapid response to COVID-19 was made possible by decades of investment in basic science, especially on mechanisms of immunity and tools to explore the immune response against infectious diseases. Prior progress in sequencing and bioinformatics, recombinant DNA and genetic engineering, structure-based vaccine design, protein engineering of self-assembling nanoparticles, nucleic acid and vector-based vaccines, vaccine formulation, and rapid DNA synthesis and genetic engineering helped accelerate the development of COVID-19 vaccines [7].

**Applying lessons from other vaccines**. Vaccines against the Severe Acute Respiratory Syndrome (SARS) and Middle East Respiratory Syndrome (MERS) coronaviruses have been under development since those pathogens emerged, both as targets in their own right and as “prototype pathogens” for emerging coronaviruses [8]. Once the SARS-CoV-2 sequence was identified and shared, experience gained with SARS and MERS candidates was applied to development of COVID-19 vaccines [9], [10]. Structure-guided immunogen design to stabilize the SARS-CoV-2 spike protein in pre-fusion conformation also drew on experience gained from immunogen design for respiratory syncytial virus fusion glycoproteins [11].

**Innovative vaccine platforms for rapid response**. COVID-19 accelerated the development of technology platforms capable of generating novel vaccine constructs rapidly in response to new threats. No single platform has emerged as a clear “winner” from COVID-19; rather, the global response to the pandemic has demonstrated the value of having a diversity of platforms, each with attributes that determine their suitability for use in different contexts.

*Nucleic acid vaccines,* including RNA-based and DNA-based vaccines, have been in development for many years [12]. Compared to other types of vaccines, RNA-based vaccines can be designed rapidly, have high potency, and are simpler to manufacture. In spite of these advantages, no RNA-based vaccines had been licensed for use in humans prior to COVID-19, due in part to the need to improve thermostability, immunogenicity, and tolerability [13]. In response to the pandemic, highly purified mRNA vaccines formulated in lipid nanoparticles from Pfizer/BioNTech (Comirnaty®) and Moderna (Spikevax™) were among the first vaccines to achieve Emergency Use Authorization (EUA), widespread deployment, and licensure [14], [15], [16], [17]. While improvements are still needed, especially in thermostability, participants spoke of using RNA vaccines to target other viruses that have complex antigens such as respiratory syncytial virus and herpesvirus; to tackle non-viral targets such as bacteria or protozoa; to replace existing vaccines such as those for influenza or rabies; and to be deployed in combination with other vaccine types in heterologous prime-boost strategies. RNA-based platforms are also being considered for *in vivo* synthesis of antibodies for passive immunization or immunotherapy. DNA-based vaccines are also in development for many pathogens, including Zika virus and SARS-CoV-2 [13], [18]. DNA vaccines can be administered using electroporation devices that yield increased immunogenicity and consistency, but this approach currently has limited real-world applicability [13], [19].

*Viral-vectored vaccine platforms* include replication-competent viral vectors, such as vesicular stomatitis virus, measles, or cytomegalovirus, and replication-incompetent vectors based on viruses such as vaccinia or adenovirus. Viral-vectored vaccines have now been licensed for Ebola virus disease and COVID-19. They are generally well-tolerated and do not require adjuvants but can present novel challenges in process development, manufacturing, or scale-up. Viral-vectored COVID-19 vaccines from Gamaleya, Oxford-AstraZeneca, CanSino, and Janssen have now been approved for emergency use in many countries [15]. As presented at GVIRF, the immune response to the Oxford-AstraZeneca COVID-19 vaccine, Vaxzevria™, is primarily to the delivered antigen rather than the non-replicating simian adenovirus vector, and anti-vector immunity has a negligible effect even when a second dose is delivered 28 days after the first dose [20].

*Adjuvants*. Adjuvants can be considered platform technologies because they can increase antigen uptake and immune stimulation by a variety of antigens, thereby enhancing immune responses. The choice of an adjuvant is a critical product development decision for subunit-based vaccines and may depend on access to intellectual property, raw material availability, cost and ease of production, and the clinical evidence needed. For future pandemics, adjuvant components could be stockpiled to prevent shortages of critical raw materials.

**Investments in preparedness.** As recognized by multiple meeting participants, the ability to respond in an effective and timely manner to a global public health emergency such as COVID-19 requires not only a preceding investment in a robust and diverse scientific foundation but also the flexibility and willingness to pursue in a timely manner - even at considerable risk - further R&D efforts as well as support manufacturing at scale. Not surprisingly, this role has often defaulted to large entities, and government agencies have clearly played a role in supporting this approach. Interestingly, however, non-government agencies have also emerged to play this role.

The Coalition for Epidemic Preparedness Innovations (CEPI) illustrates the value of both preparedness and the prototype pathogen approach. Created after the 2014/2015 Ebola virus disease outbreak in west Africa, CEPI’s mission is to accelerate the development of vaccines against emerging infectious diseases and enable equitable access to these vaccines during outbreaks [21]. CEPI has funded the development of vaccines against MERS and invested in platform technologies for rapid response. Upon emergence of SARS-CoV-2, CEPI rapidly redirected its investments to develop novel coronavirus vaccines. It has taken a portfolio approach to maximize the chances of success and to advance vaccines with a diversity of product attributes, such as cold chain requirements and doses per course. As of GVIRF 2021, CEPI had supported development of 14 COVID-19 vaccines using a variety of platforms, including the Oxford/AstraZeneca and Moderna vaccines, which have obtained Emergency Use Listing (EUL) from the WHO [22]. To enable equitable access, CEPI has also helped to build manufacturing capacity, investing at risk in parallel with clinical trials to enable the fastest response possible. Similar at-risk investments by national governments to accelerate R&D and scale manufacturing across multiple vaccines exemplify the strategic approach necessary to create an environment that enables global access.

**Leadership and coordination for preparedness and response**. WHO, as the central coordinator in preventing international spread of disease, established the *R&D Blueprint for Action to Prevent Epidemics* to prioritize diseases for R&D in emergency contexts and to rapidly activate R&D activities during epidemics [23]. The Blueprint has facilitated responses to Ebola virus disease as well as COVID-19. For COVID-19, WHO has contributed to the global research roadmap, monitored the landscape of candidate vaccines, proposed criteria for prioritizing among candidate vaccines, defined a target product profile that describes desirable attributes from a global public health perspective, supported information sharing, and facilitated clinical trial and observational study design and execution—including human challenge studies [24]. Ongoing WHO priorities include a risk assessment framework for SARS-CoV-2 variants, regulatory alignment and coordinated approaches for vaccines targeting new variants, and correlates of protection for licensure of future COVID-19 vaccines.

**Partnerships for Global Equitable Access**. COVAX is the vaccines “pillar” of the Access to COVID-19 Tools Accelerator. It incentivizes rapid manufacturing, fair distribution, and equitable delivery of COVID-19 vaccines. As a partnership led by CEPI, WHO, and Gavi, the Vaccine Alliance, COVAX is serving 190 participating economies, including 92 low- and middle-income countries that would otherwise face great difficulties securing vaccines. COVAX started vaccine deliveries in early 2021 and as of December 2021 forecasted that 1.4 billion doses of COVID-19 vaccines would be available through COVAX by the end of 2021 [25]. In spite of global collaboration to broaden access to COVID-19 vaccines, low-resource settings remain poorly served. Challenges have included unpredictable supply as well as product attributes poorly suited to delivery in low-resource settings, such as the need for ultra-low temperature storage. GVIRF speakers called for purposeful, coordinated action to scale up production and distribution of COVID-19 vaccines. As one speaker noted, “Ethically, it’s the right thing to do. Economically, it’s the self-interested thing to do, as prolonging the epidemic will deepen its damage. Biologically, it’s the necessary thing to do, or the virus will evolve and escape our countermeasures.”

## 3 Lessons from COVID-19

The COVID-19 pandemic has changed the global landscape of vaccines and immunization research. Participants put forward the following lessons for pandemic preparedness as well as for control of endemic disease.

**Need for a coordinated approach**. Speakers emphasized that pandemic preparedness and response, including to emerging variants, requires a coordinated, global approach with increased health systems support and investment in research in inter-pandemic periods. CEPI and others aspire to respond to future pandemics with safe, effective vaccines within 100 days [26]. To do so will require preparation in inter-pandemic times by: understanding relevant immune responses and how to modulate them; improving global surveillance and epidemiology; defining what clinical data are needed for each disease and vaccine; and building regional clinical trial, manufacturing, regulatory, and distribution capacity.

**Invest in research capability**. COVID-19 has shown that robust, ongoing, regional and international R&D programs in emerging infectious diseases, models and reagents, and vaccine platform technologies are central to preparedness and response. Capacity is needed in: fundamental and translational vaccine research; comprehensive surveillance and notification mechanisms; and “real-time” research to refine vaccine approaches and clinical trial strategies, especially for emerging variants. Systems for data, sample, and pathogen sharing will enable collaboration and accelerate progress.

**Target prototype pathogens**. While the R&D Blueprint prioritizes a handful of pathogens, some speakers advocated for a more expansive approach that systematically targets the 25 or more viral families known to infect humans. This approach would choose prototype viruses representing each family and advance vaccine candidates for each one through Phase 1 clinical trials. Vaccines against additional viruses with pandemic potential could be advanced through animal testing [27], [28].

**Engage industry.** The public sector has cooperated with industry for COVID-19 vaccine R&D in many ways. It has created research initiatives that foster the development of new vaccine platform technologies, directly supported companies engaged in vaccine development, engaged with companies that have the capability to develop or manufacture vaccines to meet local and global demand, funded clinical development, and shortened timelines for regulatory review [29], [30]. For future emergencies, speakers emphasized the importance of incentivizing international collaboration and cooperation in clinical trials, ensuring analysis of correlates of protection is incorporated into clinical trial plans, and establishing regulatory processes that routinely work faster without sacrificing safety.

**Improve manufacturing readiness**. Responses to Ebola virus disease outbreaks and the COVID-19 pandemic have encountered manufacturing-related challenges, including the variations in regulatory requirements, complexity of technology transfer, insufficient supply of raw materials, and limited global manufacturing capacity that is concentrated in a few regions. Partly due to these challenges, COVID-19 vaccines were rolled-out first in the higher income countries where vaccine production capacity is concentrated [31].

Speakers called for a global, proactive approach to overcome these challenges and combat current and future epidemics. Readiness will require manufacturing technologies that are both flexible, to rapidly respond to an emerging disease, and scalable, since the scope of emergencies can vary widely. Nucleic acid vaccine platforms create opportunities to build capacity in regions that currently have no or limited vaccine manufacturing capacity. An additional manufacturing approach was presented: intended to improve access to affordable vaccines, this method combines bioprocess intensification with modular micro-facilities that could be deployed where needed to produce viral-vectored, inactivated, or live-attenuated vaccines [32].

For ongoing readiness, manufacturing facilities must be maintained and run continually (be “ever-warm”) for rapid deployment when an emergency strikes. These facilities could manufacture vaccines for emergency response stockpiles between emergencies, then rapidly initiate or scale-up manufacturing when an outbreak occurs. Between emergencies, they must serve markets that are large enough to be financially sustainable; during an emergency, they must have sufficient capacity to rapidly satisfy domestic needs. Innovative financing and business models will be needed to make this possible. Innovative approaches that encourage and enable technology transfer are also needed. These go beyond overcoming intellectual property barriers, and include facilitating the investment of time, resources, and know-how.

Global preparedness includes addressing the geographic imbalances in manufacturing capacity and the skilled personnel needed to support high-quality manufacturing capability. Distributing manufacturing capacity globally would reduce the risk that some populations will have limited access due to supply chain failures or export restrictions. Multiple regional hubs for vaccine production have been announced since GVIRF 2021, a sign of growing momentum for regional vaccine manufacturing [33], [34]. By producing regionally important vaccines and transferring these technologies to existing and new manufacturers, these hubs could help to address current vaccine inequities and prepare for future threats.

**Improve vaccine administration**. The COVID-19 pandemic has increased investment in innovations to improve vaccine administration, such as microarray patches, blow-fill-seal devices, and multi-dose pouches [35], [36], [37], [38]. These innovations have the potential to increase access to COVID-19 vaccines as well as other essential vaccines in use or in development.

Innovations in administration must consider key aspects of manufacture, adoption, and use in order to achieve maximum impact. To ensure that new administration technologies are fit for purpose, potential users must be involved in the development process. To ensure availability of such technologies, it will be important to consider where they will be manufactured, what data regulators and policymakers will require, what production throughput will be possible, effects on manufacturing and delivery costs per dose of vaccine, and who will procure and pay for vaccines that use the new technology. To promote uptake and avoid contributing to vaccine hesitancy, community engagement will be needed to build acceptance and demand.

Because these processes take time, innovations to improve vaccine administration should not wait until there is an emergency but instead be part of coordinated, ongoing emergency preparedness efforts and initiatives to improve access to other vaccines. One such initiative is the Vaccine Innovation Prioritization Strategy (VIPS), a collaboration between Gavi, WHO, the Bill & Melinda Gates Foundation, UNICEF, and PATH. To improve vaccine administration, VIPS has assessed innovative technologies and conducted stakeholder consultations to develop consensus on what new vaccine technologies are needed to increase coverage. VIPS partners are now accelerating the advancement of selected new technologies [39], [40], [41].

**Many reasons for optimism**. Although the pandemic continues to take its toll and the virus continues to evolve, speakers found reasons to be optimistic. In their view, COVID-19 will raise the profile of vaccination and strengthen delivery systems globally as the largest vaccination program in history unfolds. COVID-19 vaccination will expand immunization programs, which typically focus on infants, to benefit people of all ages. Tools and approaches for delivering COVID-19 vaccines, such as digital immunization registries and catch-up vaccination campaigns, will help to improve access to other vaccines and health interventions. Investments in new technologies and improving existing platforms will lower manufacturing costs and reduce prices. Advances and lessons from COVID-19 are will accelerate progress against other diseases, including longstanding challenges such as human immunodeficiency virus (HIV), tuberculosis, and malaria. Health security concerns will drive regional collaboration and capacity building, including investments to expand surveillance, sequencing, and manufacturing capacity. Although built to improve regional health security, such capacity would be used between emergencies to produce vaccines of national or regional importance. Finally, stakeholders troubled by inequitable access to vaccines are calling for global solidarity, on the basis that, “No one is safe until every-one is safe.”[42] This shift in perspective would help ensure that in the future delayed access for low-income countries will no longer be tolerated.

## 4 Broader progress since GVIRF 2018

Vaccine and immunization research has progressed in many ways aside from COVID-19. Presenters reviewed notable developments and highlighted data gaps across the continuum from research to implementation.

### 4.1 Research

*Controlled human infection models (CHIMs)* have been valuable tools for: understanding human-pathogen interactions and vaccine immunity; informing vaccine development; and providing evidence to support regulatory review, WHO prequalification, and vaccine introduction to endemic countries [43], [44]. Because they deliberately expose trial subjects to infection, CHIMs raise important ethical and social questions, particularly in low- and middle-income countries where risks, burden, and benefits may be different than in high-income countries [45]. As illustrated by malaria challenge studies, CHIMs can be conducted to high ethical standards in low-resource settings and yield insights only obtainable in endemic areas [46], [47]. After carefully addressing technical and ethical considerations, researchers in the United Kingdom have established a SARS-CoV-2 human challenge research program to facilitate basic research and vaccine development and evaluation [48], [49].

*Knowledge gaps at both ends of the lifespan*. In newborns, bacille Calmette-Guérin (BCG) vaccines for tuberculosis provide broad and immediate protection against a range of other pathogens and reduce infectious disease morbidity and mortality. These non-specific benefits are not seen in all settings, and their mechanism of action remains poorly understood [50]. Increasing *T*-cell senescence and other age-related changes in immunity are also poorly understood, and little is known about ways to reduce biological aging and preserve immune function [51].

### 4.2 Vaccine R&D

*Tuberculosis* remains a leading cause of death, with 1.6 million people dying of the disease in 2021 [52], [53]. Current or previous tuberculosis raises the risk of COVID-19 mortality, and COVID-19-related reductions in detection and treatment could increase tuberculosis deaths by 20 % over the next five years [54], [55]. Vaccine candidates to prevent both infection and disease are being assessed in a variety of populations, with two notable efficacy results. A Phase 2 trial evaluated revaccination of adolescents with BCG, a tuberculosis vaccine widely used in infants. This trial found positive efficacy in preventing sustained infection, and a confirmatory trial is underway in South Africa [56], [57]. A Phase 2b trial of M72/AS01_E_ in infected adults demonstrated 49.7 % protection against progression to active disease [58], [59]. A Phase 2 trial of M72/AS01_E_ in persons living with HIV is underway, and a pivotal efficacy trial is in the planning stages [60].

*HIV.* Two vaccine trials have been halted due to lack of efficacy in preventing HIV infection. The Phase 2b/3 HVTN702 trial evaluated a combination regimen of ALVAC-HIV and MF59-adjuvanted, bivalent subtype C gp120 [61]. The Phase 2b HVTN705 trial evaluated a combination regimen of Ad26.Mos4.HIV (an adenovirus-vectored vaccine) and aluminum phosphate-adjuvanted, trimeric, clade C gp140 [62], [63]. Serious safety issues were not noted in either trial. The Phase 3 HVTN706 trial is underway and expected to yield results in 2024. This trial is evaluating a combination regimen of Ad26.Mos4.HIV and bivalent clade C and Mosaic gp140 [64]. HIV vaccine candidates now in Phase 1 testing include novel approaches such as germline targeting and native-like Env trimers [65], [66], [67].

Phase 2b trials evaluating VRC01, a broadly neutralizing antibody (bnAb) to HIV-1, showed that antibody infusions could prevent infection by some HIV-1 strains. While VRC01 did not prevent HIV-1 acquisition overall, it blocked infection by HIV strains sensitive to the antibody *in vitro*. Thus, these trials provided proof-of-concept that bnAbs can be effective in HIV prophylaxis. Because protection depends on the sensitivity of HIV-1 strains circulating in the target population, antibody combinations may be needed for broad efficacy [68].

*Malaria*. The most advanced malaria vaccine candidate, RTS,S/AS01 (Mosquirix™), which showed modest efficacy in a large multicenter clinical trial, received a positive opinion from the European Medicines Agency, and in 2019 entered into a pilot implementation program in Ghana, Kenya and Malawi [69]. This vaccine has since been recommended for widespread use among children in sub-Saharan Africa and in other regions with moderate to high *Plasmodium falciparum* transmission [70]. Nevertheless, there is widespread recognition of the need for malaria vaccines with greater efficacy and producing more durable protection, and for data on their potential impact when delivered in the context of ongoing malaria control efforts. Clinical and preclinical malaria vaccine development remains robust, with over 100 malaria vaccine trials registered in the past decade and vaccines in the pipeline that target diverse lifecycle stages of malaria parasites using a variety of technology platforms. Human malaria challenge models are an important advance, enabling at an early stage of clinical development efficient evaluation of immunogens, adjuvants, novel vaccine constructs, and monoclonal antibodies [46]. Moving forward, it will be important to apply the lessons learned from RTS,S/AS01 and COVID-19 vaccine development and make appropriate resourcing available to enable the timely development, registration, and implementation of novel malaria vaccine candidates and vaccination strategies.

*Influenza.* Cross-protective, “universal” influenza vaccines will need to elicit strong protection against a broad spectrum of influenza strains, have acceptable safety profiles, and overcome regulatory and implementation challenges [71]. Current approaches to improving cross-protection by influenza vaccines include adjuvants, whole virion vaccines, platform combinations, antigen optimization, and combination vaccines [72]. Lessons from COVID-19 vaccine development in adjuvants and mRNA vaccines are being explored for applicability to influenza.

*Human papillomavirus (HPV).* Effectiveness studies and post-hoc analysis of randomized, controlled trials in India and Costa Rica have suggested that long-term protection may result from a single dose of HPV vaccine [73], [74], [75], [76]. Additional research is underway to provide more robust data on single-dose regimens and to inform HPV vaccination policy [77], [78].

*Poliovirus.* A novel oral poliomyelitis type 2 vaccine (nOPV2) was granted EUL by WHO in November 2020 [79]. Due to its enhanced genetic stability, nOPV2 has a lower risk of seeding vaccine-derived polio outbreaks. Provided that it meets safety and genetic stability criteria during an initial use period, nOPV2 could become the vaccine of choice for controlling poliomyelitis type 2 outbreaks. Since the time of the GVIRF meeting in 2021, broad uptake of nOPV2 has occurred with over 500 million doses administered in 23 countries. Monitoring of safety and effectiveness is underway.

*Other priority diseases.* Clinical studies have been launched for multiple vaccines that target schistosomiasis [80], [81] or invasive non-typhoidal salmonella [82], [83], [84]. Two typhoid conjugate vaccines have been pre-qualified by WHO [85] and programs using these vaccines are learning how to integrate them into broader disease control programs and conducting post-introduction effectiveness studies.

### 4.3 Vaccine uptake and demand

Research on the behavioral and social determinants of vaccination has documented the many reasons why people do or do not receive life-saving vaccines. These include: what people think and feel about vaccines; social norms; hesitancy or motivation to vaccinate; and practical issues such as access to high-quality, convenient services [86]. Interventions should be informed by surveys that uncover the specific challenges or concerns of a given community. Asking, “Would you accept the vaccine?” is not enough; action-oriented questions such as, “What information do you want? From whom?” are more useful. Key data users should be involved in survey development to ensure that the data collected will inform communication strategies. Engagement with communities should be reciprocal: relationships built by understanding and acting on a community’s priorities can then enable resilient, sustained uptake of vaccines [87]. This understanding has contributed to a toolkit of approaches to increasing vaccine uptake [88].

### 4.4 Priorities for investment by the public and private sectors

Given the central role of industry in developing and producing vaccines, achieving public health goals requires priorities and incentives that are aligned across the public and private sectors. The need is real: a survey of 20 major pharmaceutical companies found that none were conducting R&D for 10 out of 16 emerging infectious diseases, and over 60 % of their late-stage development projects were not supported by access plans [89]. GVIRF participants commented that markets are perceived as too fragile and incentives are too inconsistent to justify investment, leading to these unmet needs. Improving alignment could enable vaccine development for a wide range of diseases and improve affordability and access for existing vaccines.

Within the public sector, aligning priorities requires agreement on what factors should drive prioritization decisions. In the past, these factors have included data on burden of disease, technical feasibility, and return on investment. Now COVID-19 has also shown the importance of regional capacity for vaccine research, development, and manufacturing to enable equitable responses to emergencies. Better alignment in public sector priorities at global and regional levels could yield more transparent policy goals, well-defined target product profiles, and concrete demand projections that lower risks for private sector investments.

Where market incentives are not sufficient to motivate private sector engagement, public–private partnerships (PPPs) have succeeded in aligning priorities across sectors and building the cross-stakeholder platforms needed to rapidly scale up vaccine implementation. CEPI is a striking example: by aligning policy positions, pooling resources for pull and push incentives, and standardizing demand assessments, they fostered vaccine R&D for emerging infectious diseases that was redeployed rapidly for COVID-19 response [90]. PPPs can also go beyond development of new interventions to: support reformulations or combination products; promote R&D with broader applications to other diseases such as new vaccine platform technologies, new adjuvants, alternative administration routes, and low-cost manufacturing; and improve access in target countries. Companies represented by the Developing Countries Vaccine Manufacturers Network have been well-positioned to partner in these PPPs to accelerate equitable access in geographies that have been historically underserved by new products.

### 4.5 Accelerating policy making

COVID-19 vaccines have been approved using new, faster regulatory mechanisms such as EUAs [91], [92]. This precedent may catalyze the use of more rapid regulatory strategies for other vaccines, enabling earlier licensure. Unless policy processes are also expedited, however, this will lead to an implementation gap, rather than more rapid impact.

To shorten lags between vaccine licensure and implementation, WHO is piloting Evidence Considerations for Vaccine Policy (ECVP) briefs. ECVPs for new vaccines in priority disease areas would be established early in clinical development—prior to the design of Phase 3 trials—to provide guidance on the evidence needed for WHO and policy-decision making. ECVPs would expedite policy processes and reduce uncertainties for manufacturers by ensuring that vaccines are programmatically relevant and ready for use; addressing country-to-country differences in epidemiology, needs, and priorities; encouraging dialogue and alignment between regulators and policy makers; and incorporating the perspectives of financing and procurement agencies. A WHO ECVP for tuberculosis vaccines for adolescents and adults will pilot this concept as the first test case.

In order to succeed, ECVP and other approaches to establishing preferred policy profiles must include country-level stakeholders, program managers, and end-users.

## 5 Immunization Agenda 2030

GVAP defined for the first time a global strategy for R&D in immunization, focusing attention on product pipelines and barriers to impact [93]. Its successor, IA2030, emphasizes equity and aspires to “A world where everyone, everywhere, at every age, fully benefits from vaccines for good health and well-being.” [3] Research and innovation are essential to achieving this vision.

Developing new vaccines and technologies and improving existing products and services for immunization programs will remain a focus in this decade. To guide investment and smooth the path to impact, the IA2030 Research and Innovation agenda aims to improve alignment between country priorities and the global mechanisms that foster innovation. IA2030 also aims to strengthen national and regional capacity to: identify priorities for innovation; create and manage innovation; evaluate promising innovations; and scale up innovative products, services, and practices as appropriate on the basis of the best available evidence [94].

At GVIRF, speakers discussed how research and innovation will be needed to recover from the COVID-19 pandemic and to achieve the goals of IA2030. COVID-19 has disrupted routine immunization, and by 2021, the number of children who were un- and under-vaccinated had risen to 25 million. The number of “zero-dose children”—those who did not receive their first dose of diphtheria, tetanus, and pertussis-containing vaccine—increased to 18 million [95]. These children disproportionately live in Africa and in conflict-affected countries. Speakers emphasized that equity, a central aim of IA2030, requires both equitable access to vaccines and resilient health systems that are prepared for the next pandemic.

## 6 Conclusion

Vaccine and immunization research has progressed on many fronts since GVIRF 2018, and remains the foundation on which success will be built going forward. The COVID-19 pandemic, however, has triggered extraordinary achievements and the potential for a new way of thinking about product development. Advances in vaccine R&D and manufacturing, in combination with new paradigms in international cooperation and regional investments, will have benefits across the vaccine ecosystem for years to come. Equity is the rallying cry for immunization this decade, and research and innovation will pay an important role in improving equity in access to vaccines. Partnerships between national, regional, and global stakeholders will be needed to inform priorities and accelerate progress in research and innovation.

The momentum toward development of SARS-CoV-2 vaccines and implementation of COVID-19 vaccination programs can and must contribute to developing vaccines against other burdensome infectious diseases, pursuing the vision of IA2030 in which no-one is left behind, and fully realizing the value of vaccines. As the central forum for the global vaccine and immunization research community, GVIRF will take stock again in 2023.

## Data Availability

No data was used for the research described in the article.

## References

[1] Sim S.Y., Watts E., Constenla D., Brenzel L., Patenaude B.N. (2020). Return on Investment from Immunization Against 10 Pathogens In 94 Low- and Middle-Income Countries, 2011–30. Health Aff (Millwood).

[2] Chang A.Y., Riumallo-Herl C., Perales N.A., Clark S., Clark A., Constenla D. (2018). The Equity Impact Vaccines May Have On Averting Deaths And Medical Impoverishment In Developing Countries. Health Aff (Millwood).

[3] Immunization Agenda 2030. Immunization Agenda 2030: A global strategy to leave no one behind. 2021. https://www.immunizationagenda2030.org/ [accessed December 30, 2021].

[4] Vanderslott S, Dadonaite B, Roser M. Vaccination. OurWorldinData.org, 2013. Graphic courtesy of Melanie Saville of the Coalition for Epidemic Preparedness Innovations. https://ourworldindata.org/vaccination.

[5] Joyce M.G., Chen W.H., Sankhala R.S., Hajduczki A., Thomas P.V., Choe M. (2021). SARS-CoV-2 ferritin nanoparticle vaccines elicit broad SARS coronavirus immunogenicity. Cell Rep.

[6] Rogers J. Going universal: The search for an all-in-one coronavirus vaccine. Coalition for Epidemic Preparedness Innovations, 2021. https://cepi.net/news_cepi/going-universal-the-search-for-an-all-in-one-coronavirus-vaccine/ [accessed February 2, 2022].

[7] Rappuoli R., De Gregorio E., Del Giudice G., Phogat S., Pecetta S., Pizza M. (2021). Vaccinology in the post-COVID-19 era. Proc Natl Acad Sci U S A.

[8] World Health Organization. Overview of the types/classes of candidate vaccines against MERS-CoV. 2021. https://www.who.int/publications/m/item/overview-of-the-types-classes-of-candidate-vaccines-against-mers-cov [accessed September 22, 2021].

[9] Corbett K.S., Edwards D.K., Leist S.R., Abiona O.M., Boyoglu-Barnum S., Gillespie R.A. (2020). SARS-CoV-2 mRNA vaccine design enabled by prototype pathogen preparedness. Nature.

[10] Wrapp D., Wang N., Corbett K.S., Goldsmith J.A., Hsieh C.L., Abiona O. (2020). Cryo-EM structure of the 2019-nCoV spike in the prefusion conformation. Science.

[11] McLellan J.S., Chen M., Leung S., Graepel K.W., Du X., Yang Y. (2013). Structure of RSV fusion glycoprotein trimer bound to a prefusion-specific neutralizing antibody. Science.

[12] Qin F., Xia F., Chen H., Cui B., Feng Y., Zhang P. (2021). A Guide to Nucleic Acid Vaccines in the Prevention and Treatment of Infectious Diseases and Cancers: From Basic Principles to Current Applications. Front Cell Dev Biol.

[13] Verdecia M., Kokai-Kun J.F., Kibbey M., Acharya S., Venema J., Atouf F. (2021). COVID-19 vaccine platforms: Delivering on a promise?. Hum Vaccin Immunother.

[14] Stuart L.M. (2021). In Gratitude for mRNA Vaccines. N Engl J Med.

[15] Zimmer C, Corum J, Wee S-L. Coronavirus Vaccine Tracker. New York Times, 2021. https://www.nytimes.com/interactive/2020/science/coronavirus-vaccine-tracker.html [accessed March 19, 2021].

[16] Dolgin E. (2021). The tangled history of mRNA vaccines. Nature.

[17] Krause P.R., Fleming T.R., Peto R., Longini I.M., Figueroa J.P., Sterne J.A.C. (2021). Considerations in boosting COVID-19 vaccine immune responses. Lancet.

[18] Tebas P., Yang S., Boyer J.D., Reuschel E.L., Patel A., Christensen-Quick A. (2021). Safety and immunogenicity of INO-4800 DNA vaccine against SARS-CoV-2: A preliminary report of an open-label, Phase 1 clinical trial. EClinicalMedicine.

[19] Xu Z., Patel A., Tursi N.J., Zhu X., Muthumani K., Kulp D.W. (2020). Harnessing Recent Advances in Synthetic DNA and Electroporation Technologies for Rapid Vaccine Development Against COVID-19 and Other Emerging Infectious Diseases. Frontiers in Medical. Technology.

[20] Ramasamy M.N., Minassian A.M., Ewer K.J., Flaxman A.L., Folegatti P.M., Owens D.R. (2021). Safety and immunogenicity of ChAdOx1 nCoV-19 vaccine administered in a prime-boost regimen in young and old adults (COV002): a single-blind, randomised, controlled, phase 2/3 trial. Lancet.

[21] Gouglas D., Christodoulou M., Plotkin S.A., Hatchett R. (2019). CEPI: Driving Progress Toward Epidemic Preparedness and Response. Epidemiol Rev.

[22] Coalition for Epidemic Preparedness Innovations. Our Portfolio. https://cepi.net/research_dev/our-portfolio/ [accessed November 12, 2021].

[23] World Health Organization. An R&D Blueprint for Action to Prevent Epidemics, Update 2017. 2017. https://cdn.who.int/media/docs/default-source/blue-print/an-randd-blueprint-for-action-to-prevent-epidemics-update-2017.pdf [accessed January 1, 2023].

[24] World Health Organization. COVID-19 Research and Innovation Achievements. 2021. https://cdn.who.int/media/docs/default-source/documents/r-d-blueprint-meetings/r-d-achievements-report_v42.pdf [accessed November 12, 2021].

[25] COVAX. COVAX Global Supply Forecast. 2021. https://www.gavi.org/sites/default/files/covid/covax/COVAX-Supply-Forecast.pdf [accessed January 5, 2022].

[26] Pandemic Preparedness Partnership. 100 Days Mission to Respond to Future Pandemic Threats: Reducing the impact of future pandemics by making Diagnostics, Therapeutics and Vaccines available within 100 Days. https://assets.publishing.service.gov.uk/government/uploads/system/uploads/attachment_data/file/992762/100_Days_Mission_to_respond_to_future_pandemic_threats__3_.pdf [accessed December 30, 2021].

[27] Graham B.S., Corbett K.S. (2020). Prototype pathogen approach for pandemic preparedness: world on fire. J Clin Invest.

[28] Graham B.S., Sullivan N.J. (2018). Emerging viral diseases from a vaccinology perspective: preparing for the next pandemic. Nat Immunol.

[29] Division of Research, Innovation, and Ventures: Revolutionize the Way We Prevent, Detect, and Respond to Major 21st Century Health Security Threats. U.S. Department of Health & Human Services, Biomedical Advanced Research and Development Authority. https://drive.hhs.gov/index.html [accessed February 2, 2022].

[30] World Health Organization. Establishment of a COVID-19 mRNA vaccine technology transfer hub to scale up global manufacturing: Expression of interest. 2021. https://www.who.int/news-room/articles-detail/establishment-of-a-covid-19-mrna-vaccine-technology-transfer-hub-to-scale-up-global-manufacturing [accessed February 2, 2022].

[31] Our World in Data. COVID-19 vaccine doses administered per 100 people, by income group. https://ourworldindata.org/grapher/cumulative-covid-vaccinations-income-group?country=High+income∼Low+income∼Lower+middle+income∼Upper+middle+income [accessed December 30, 2021].

[32] Batavia Biosciences. HIP-Vax: low-cost vaccine manufacturing. www.bataviabiosciences.com/viral-vaccines/technologies/hip-vax-vaccine-manufacturing/ [accessed December 30, 2021].

[33] World Health Organization. New consortium working to boost vaccine production in South Africa. MPP, WHO, AFRIGEN, BIOVAC, SAMRC, and Africa CDC sign a letter of intent towards establishing the tech transfer hub in South Africa. Geneva.2021. https://www.who.int/news/item/30-07-2021-new-consortium-working-to-boost-vaccine-production-in-south-africa [accessed January 1, 2022].

[34] Pan American Health Organization. PAHO selects centers in Argentina, Brazil to develop COVID-19 mRNA vaccines. 2021. https://www.paho.org/en/news/21-9-2021-paho-selects-centers-argentina-brazil-develop-covid-19-mrna-vaccines [accessed February 2, 2022].

[35] US Department of Health & Human Services. BARDA establishes four new partnerships to explore innovative vaccine delivery technologies. 2020. https://www.medicalcountermeasures.gov/newsroom/2020/barda-new-partnerships/ [accessed January 3, 2022].

[36] Parkins K. (2021). Covid-19 vaccine patch outshines injectable jab in pre-clinical study. Clinical Trials Arena.

[37] Integrated Project Services. Blow-Fill-Seal & Prefilled Syringes: A Solution for Complex Formulations. https://www.ipsdb.com/happenings/insights/blow-fill-seal-prefilled-syringes-a-solution-for-complex-formulations [accessed January 3, 2022].

[38] Downham M, Hesselink R. Multidose Prefilled Pouches for Pandemics. 2021. https://www.dcvmn.org/IMG/pdf/dcvmn_webinar_the_multidose_prefilled_bags_for_pandemics_08apr21.pdf [accessed January 3, 2022].

[39] Kristensen D., Giersing B., Hickling J., Kazi F., Scarna T., Kahn A.L. (2021). A global collaboration to advance vaccine product innovations - The Vaccine Innovation Prioritisation Strategy. Vaccine.

[40] Giersing B., Shah N., Kristensen D., Amorij J.P., Kahn A.L., Gandrup-Marino K. (2021). Strategies for vaccine-product innovation: Creating an enabling environment for product development to uptake in low- and middle-income countries. Vaccine.

[41] Mvundura M., Frivold C., Janik Osborne A., Soni P., Robertson J., Kumar S. (2021). Vaccine innovation prioritisation strategy: Findings from three country-stakeholder consultations on vaccine product innovations. Vaccine.

[42] von der Leyen U, Ghebreyesus TA. A global pandemic requires a world effort to end it - none of us will be safe until everyone is safe. The Telegraph.2020. https://www.telegraph.co.uk/global-health/science-and-disease/global-pandemic-requires-world-effort-end-none-us-will-safe/ [accessed February 2, 2022].

[43] Sekhar A., Kang G. (2020). Human challenge trials in vaccine development. Semin Immunol.

[44] Paterson S., Kar S., Ung S.K., Gardener Z., Bergstrom E., Ascough S. (2021). Innate-like Gene Expression of Lung-resident Memory CD8+ T-cells During Experimental Human Influenza: A Clinical Study. Am J Respir Crit Care Med.

[45] Jamrozik E., Selgelid M.J. (2020). Ethical issues surrounding controlled human infection challenge studies in endemic low-and middle-income countries. Bioethics.

[46] Chi P.C., Owino E.A., Jao I., Olewe F., Ogutu B., Bejon P. (2021). Understanding the benefits and burdens associated with a malaria human infection study in Kenya: experiences of study volunteers and other stakeholders. Trials.

[47] Kapulu M.C., Njuguna P., Hamaluba M., Kimani D., Ngoi J.M., Musembi J. (2021). Safety and PCR monitoring in 161 semi-immune Kenyan adults following controlled human malaria infection. JCI. Insight.

[48] Rapeport G., Smith E., Gilbert A., Catchpole A., McShane H., Chiu C. (2021). SARS-CoV-2 Human Challenge Studies - Establishing the Model during an Evolving Pandemic. N Engl J Med.

[49] Jamrozik E., Littler K., Bull S., Emerson C., Kang G., Kapulu M. (2021). Key criteria for the ethical acceptability of COVID-19 human challenge studies: Report of a WHO Working Group. Vaccine.

[50] Prentice S., Dockrell H.M. (2021). BCG Specific and Nonspecific Effects: Different Questions. Similar Challenges J Infect Dis.

[51] Palacios-Pedrero M.A., Osterhaus A., Becker T., Elbahesh H., Rimmelzwaan G.F., Saletti G. (2021). Aging and Options to Halt Declining Immunity to Virus Infections. Front Immunol.

[52] World Health Organization. Global Tuberculosis Report. 2022. https://apps.who.int/iris/rest/bitstreams/1474924/retrieve [accessed January 1, 2023].

[53] World Health Organization. Global Tuberculosis Report. 2021. https://apps.who.int/iris/rest/bitstreams/1379788/retrieve [accessed December 28, 2021].

[54] Hogan A.B., Jewell B.L., Sherrard-Smith E., Vesga J.F., Watson O.J., Whittaker C. (2020). Potential impact of the COVID-19 pandemic on HIV, tuberculosis, and malaria in low-income and middle-income countries: a modelling study. Lancet Glob Health.

[55] Western Cape Department of Health in collaboration with the National Institute for Communicable Diseases SA. Risk Factors for Coronavirus Disease 2019 (COVID-19) Death in a Population Cohort Study from the Western Cape Province, South Africa. Clin Infect Dis. 2021;73:e2005-e15. DOI: 10.1093/cid/ciaa1198. 10.1093/cid/ciaa1198 PMC7499501 32860699

[56] Nemes E., Geldenhuys H., Rozot V., Rutkowski K.T., Ratangee F., Bilek N. (2018). Prevention of M. tuberculosis Infection with H4: IC31 Vaccine or BCG Revaccination. N Engl J Med.

[57] Bill & Melinda Gates Medical Research Institute. BCG Revaccination of Healthy Adolescents for the Prevention of Mycobacterium Tuberculosis Sustained Infection. 2021. https://clinicaltrials.gov/ct2/show/NCT04152161 [accessed December 28, 2021].

[58] Tait D.R., Hatherill M., Van Der Meeren O., Ginsberg A.M., Van Brakel E., Salaun B. (2019). Final Analysis of a Trial of M72/AS01E Vaccine to Prevent Tuberculosis. N Engl J Med.

[59] Van Der Meeren O., Hatherill M., Nduba V., Wilkinson R.J., Muyoyeta M., Van Brakel E. (2018). Phase 2b Controlled Trial of M72/AS01E Vaccine to Prevent Tuberculosis. N Engl J Med.

[60] Bill & Melinda Gates Medical Research Institute. Safety and Immunogenicity of a Mycobacterium Tuberculosis Vaccine M72/AS01E in Participants With Well-controlled HIV (MESA-TB). 2021. https://www.clinicaltrials.gov/ct2/show/NCT04556981 [accessed December 28, 2021].

[61] Gray G.E., Bekker L.G., Laher F., Malahleha M., Allen M., Moodie Z. (2021). Vaccine Efficacy of ALVAC-HIV and Bivalent Subtype C gp120-MF59 in Adults. N Engl J Med.

[62] Janssen Vaccines & Prevention B.V. A Study to Assess the Efficacy of a Heterologous Prime/Boost Vaccine Regimen of Ad26.Mos4.HIV and Aluminum Phosphate-Adjuvanted Clade C gp140 in Preventing Human Immunodeficiency Virus (HIV) -1 Infection in Women in Sub-Saharan Africa. 2021. https://clinicaltrials.gov/ct2/show/NCT03060629 [accessed December 28, 2021].

[63] International AIDS Vaccine Initiative. IAVI statement on results from Phase IIb Imbokodo HIV vaccine clinical trial. https://www.iavi.org/news-resources/features/iavi-statement-on-results-from-phase-iib-imbokodo-hiv-vaccine-clinical-trial [accessed December 28, 2021].

[64] Janssen Vaccines & Prevention B.V. A Study of Heterologous Vaccine Regimen of Adenovirus Serotype 26 Mosaic4 Human Immunodeficiency Virus(Ad26.Mos4.HIV), Adjuvanted Clade C gp140 and Mosaic gp140 to Prevent HIV-1 Infection Among Cis-gender Men and Transgender Individuals Who Have Sex With Cis-gender Men and/or Transgender Individuals (MOSAICO). 2021. https://clinicaltrials.gov/ct2/show/NCT03964415 [accessed December 30, 2021].

[65] International AIDS Vaccine Initiative. A Phase I Trial to Evaluate the Safety and Immunogenicity of eOD-GT8 60mer Vaccine, Adjuvanted. 2021. https://clinicaltrials.gov/ct2/show/NCT03547245 [accessed February 7, 2022].

[66] International AIDS Vaccine Initiative, Scripps Research. Fact sheet: Understanding the results from IAVI G001 presented at HIV R4P / Virtual 2021. https://www.iavi.org/images/phocadownload/IAVI-G001-Fact-Sheet.pdf [accessed February 7, 2022].

[67] International AIDS Vaccine Initiative. A Randomized, Double-blinded, Placebo-controlled, Dose-escalation Phase 1 Clinical Trial to Evaluate the Safety and Immunogenicity of Recombinant HIV Envelope Protein BG505 SOSIP.664 gp140 Vaccine, Adjuvanted, in Healthy, HIV-1 Uninfected Adults. 2021. https://clinicaltrials.gov/ct2/show/NCT03699241 [accessed February 7, 2022].

[68] Corey L., Gilbert P.B., Juraska M., Montefiori D.C., Morris L., Karuna S.T. (2021). Two Randomized Trials of Neutralizing Antibodies to Prevent HIV-1 Acquisition. N Engl J Med.

[69] Meeting of the Strategic Advisory Group of Experts on Immunization, October 2021 - conclusions and recommendations. Wkly Epidemiol Rec. 2021;96:613-32, https://apps.who.int/iris/bitstream/handle/10665/350649/WER9650-eng-fre.pdf [accessed December 31, 2021].

[70] World Health Organization. WHO recommends groundbreaking malaria vaccine for children at risk: Historic RTS,S/AS01 recommendation can reinvigorate the fight against malaria. 2021. https://www.who.int/news/item/06-10-2021-who-recommends-groundbreaking-malaria-vaccine-for-children-at-risk [accessed February 7, 2022].

[71] World Health Organization (WHO). WHO Preferred Product Characteristics for Next Generation Influenza Vaccines. 2017. https://apps.who.int/iris/bitstream/handle/10665/258767/9789241512466-eng.pdf [accessed December 31, 2021].

[72] Wei C.J., Crank M.C., Shiver J., Graham B.S., Mascola J.R., Nabel G.J. (2020). Next-generation influenza vaccines: opportunities and challenges. Nat Rev Drug Discov.

[73] Kreimer A.R., Sampson J.N., Porras C., Schiller J.T., Kemp T., Herrero R. (2020). Evaluation of Durability of a Single Dose of the Bivalent HPV Vaccine: The CVT Trial. J Natl Cancer Inst.

[74] Sankaranarayanan R., Joshi S., Muwonge R., Esmy P.O., Basu P., Prabhu P. (2018). Can a single dose of human papillomavirus (HPV) vaccine prevent cervical cancer? Early findings from an Indian study. Vaccine.

[75] Markowitz L.E., Drolet M., Perez N., Jit M., Brisson M. (2018). Human papillomavirus vaccine effectiveness by number of doses: Systematic review of data from national immunization programs. Vaccine.

[76] Basu P., Malvi S.G., Joshi S., Bhatla N., Muwonge R., Lucas E. (2021). Vaccine efficacy against persistent human papillomavirus (HPV) 16/18 infection at 10 years after one, two, and three doses of quadrivalent HPV vaccine in girls in India: a multicentre, prospective, cohort study. Lancet Oncol.

[77] Teppler H., Bautista O., Thomas G., Flores S., McCauley J., Luxembourg A. (2021). Design of a Phase III immunogenicity and safety study evaluating two-dose regimens of 9-valent human papillomavirus (9vHPV) vaccine with extended dosing intervals. Contemp Clin Trials.

[78] Barnabas R.V., Brown E.R., Onono M., Bukusi E.A., Njoroge B., Winer R.L. (2021). Single-dose HPV vaccination efficacy among adolescent girls and young women in Kenya (the KEN SHE Study): study protocol for a randomized controlled trial. Trials.

[79] World Health Organization. Recommendation for an Emergency Use Listing (EUL) of Novel Oral Polio Vaccine Type 2 (nOPV2) Submitted by PT Biofarma (Persero). 2020. https://extranet.who.int/pqweb/sites/default/files/documents/nOPV2_EUL_recommendation.pdf [accessed December 27, 2021].

[80] Al-Naseri A., Al-Absi S., El Ridi R., Mahana N. (2021). A comprehensive and critical overview of schistosomiasis vaccine candidates. J Parasit Dis.

[81] Molehin A.J. (2020). Schistosomiasis vaccine development: update on human clinical trials. J Biomed Sci.

[82] Sears K.T., Galen J.E., Tennant S.M. (2021). Advances in the development of Salmonella-based vaccine strategies for protection against Salmonellosis in humans. J Appl Microbiol.

[83] Marchello C.S., Fiorino F., Pettini E., Crump J.A., Vacc-i N.T.S.C.C. (2021). Incidence of non-typhoidal Salmonella invasive disease: A systematic review and meta-analysis. J Infect.

[84] Perera S.R., Sokaribo A.S., White A.P. (2021). Polysaccharide Vaccines: A Perspective on Non-Typhoidal Salmonella. Polysaccharides.

[85] World Health Organization (WHO). Comparison table of WHO prequalified typhoid conjugate vaccines (TCVs). 2021. https://apps.who.int/iris/bitstream/handle/10665/345367/WHO-IVB-2021.04-eng.pdf [accessed December 31, 2021].

[86] Larson H.J., Hartigan-Go K., de Figueiredo A. (2019). Vaccine confidence plummets in the Philippines following dengue vaccine scare: why it matters to pandemic preparedness. Hum Vaccin Immunother.

[87] Brewer N.T., Chapman G.B., Rothman A.J., Leask J., Kempe A. (2017). Increasing Vaccination: Putting Psychological Science Into Action. Psychol Sci Public Interest.

[88] World Health Organization, UNICEF. Data for action: achieving high uptake of COVID-19 vaccines: Interim guidance. 2021. https://apps.who.int/iris/rest/bitstreams/1341480/retrieve [accessed December 27, 2021].

[89] Access to Medicine Foundation. Access to Medicine Index 2021. https://accesstomedicinefoundation.org/medialibrary/resources/613f5fb390319_Access_to_Medicine_Index_2021.pdf [accessed January 1, 2023].

[90] Coalition for Epidemic Preparedness Innovations (CEPI). CEPI to fund three programmes to develop vaccines against the novel coronavirus, nCoV-2019. https://cepi.net/news_cepi/cepi-to-fund-three-programmes-to-develop-vaccines-against-the-novel-coronavirus-ncov-2019/ [accessed December 30, 2021].

[91] World Health Organization. Emergency Use Listing Procedure. 2022. https://cdn.who.int/media/docs/default-source/medicines/eulprocedure.pdf [accessed January 1, 2023].

[92] World Health Organization, International Coalition of Medicines Regulatory Authorities (ICMRA). Deep dive report on the review of provisions and procedures for emergency authorization of medical products for COVID-19 among ICMRA members - July 2021. 2021. https://www.icmra.info/drupal/sites/default/files/2021-12/eua_deep_dive_report.pdf [accessed December 30, 2021].

[93] World Health Organization Strategic Advisory Group of Experts on Immunization. The Global Vaccine Action Plan 2011-2020 Review and Lessons Learned. WHO Press, 2019. https://apps.who.int/iris/rest/bitstreams/1255869/retrieve [accessed December 27, 2021].

[94] Immunization Agenda 2030. SP7 Research and Innovation. 2021. https://www.immunizationagenda2030.org/images/documents/BLS20116_IA_Global_strategy_document_SP_7_002.pdf [accessed December 27, 2021].

[95] World Health Organization. Progress and Challenges with Achieving Universal Immunization Coverage. 2022. https://cdn.who.int/media/docs/default-source/immunization/wuenic-progress-and-challenges-15-july-2022.pdf [accessed January 1, 2023].

